# Impact of Natural Occurring *ERAP1* Single Nucleotide Polymorphisms within miRNA-Binding Sites on HCMV Infection

**DOI:** 10.3390/ijms21165861

**Published:** 2020-08-15

**Authors:** Ombretta Melaiu, Silvia D’Amico, Patrizia Tempora, Valeria Lucarini, Doriana Fruci

**Affiliations:** Department of Paediatric Haematology/Oncology and of Cell and Gene Therapy, Ospedale Pediatrico Bambino Gesù, Istituto di Ricovero e Cura a Carattere Scientifico (IRCCS), 00146 Rome, Italy; silvia.damico@opbg.net (S.D.); patrizia.tempora@opbg.net (P.T.); valeria.lucarini@opbg.net (V.L.)

**Keywords:** HCMV, microRNAs, ERAP1, single nucleotide polymorphisms, immune evasion

## Abstract

Human cytomegalovirus (HCMV) is a β-herpesvirus that causes serious problems in people with a compromised immune system, whereas it coexists asymptomatically within the host with a healthy immune system. Like other viruses, HCMV has adopted multiples strategies to manipulate the host’s immune responses. Among them, expression of viral microRNAs (miRNAs) is one of the most intriguing. HCMV miR-UL112-5p and miR-US4-1 have been found to contribute to immune evasion by targeting the endoplasmic reticulum aminopeptidase 1 (*ERAP1*), a highly polymorphic key component of antigen processing. The current incomplete picture on the interplay between viral miRNAs and host immunity implies the need to better characterize the host genetic determinants. Naturally occurring single nucleotide polymorphisms (SNPs) within the miRNA binding sites of target genes may affect miRNA–target interactions. In this review, we focus on the relevance of 3′ untranslated region (3′UTR) *ERAP1* SNPs within miRNA binding sites in modulating miRNA–mRNA interactions and the possible consequent individual susceptibility to HCMV infection. Moreover, we performed an in silico analysis using different bioinformatic algorithms to predict *ERAP1* variants with a putative powerful biological function. This evidence provides a basis to deepen the knowledge on how 3′UTR *ERAP1* variants may alter the mechanism of action of HCMV miRNAs, in order to develop targeted antiviral therapies.

## 1. Introduction

Human cytomegalovirus (HCMV) is a β-herpesvirus that persists mostly asymptomatically in infected healthy human hosts throughout their life [1], with spontaneous phases of reactivation, lytic replication, and diffusion [2]. HCMV causes several clinical syndromes in people with impaired immunity, especially in individuals subject to organ transplant, HIV-infected patients, and immunologically immature fetus [3].

**Figure 1:**
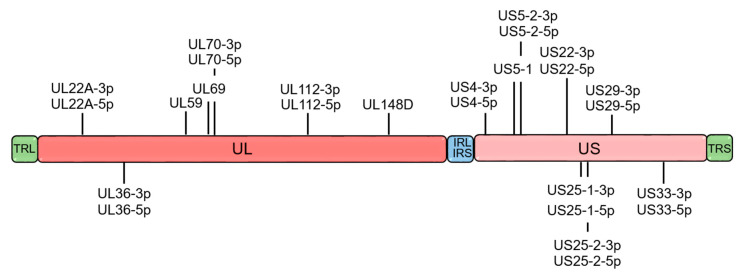
Genomic organization of human cytomegalovirus (HCMV) microRNAs (miRNAs). Localization of mature miRNAs along the linear representation of the HCMV genome divided into unique long (UL) and unique short (US) regions. These regions are flanked by terminal and internal inverted repeats (TRL, IRL, TRS, and IRS). Sense and antisense miRNA precursors are shown above and below the viral genome, respectively.

Like other viruses, HCMV has developed several strategies to affect innate and adaptive immune responses and to persist indefinitely within the host [4,5]. In essence, viral proteins are able to interfere with every step of major histocompatibility complex (MHC) class I antigen processing [6], thus indicating that presentation of viral antigens by MHC class I to cytotoxic CD8^+^ T cells is important for clearance of HCMV-infected cells. In addition to viral proteins, HCMV manipulates the host’s immune responses through microRNAs (miRNAs), a class of small non-coding RNAs of 21–25 nucleotides in length [7]. Mature miRNAs bind a sequence within the target mRNAs regulating gene expression at the post-transcriptional level. On the basis of the degree of complementarity between the seed sequence of miRNA and its binding sites on the target mRNA, the miRNA interaction can cause degradation of the target mRNA or inhibit its translation (complete complementarity and imperfect match, respectively) [8]. To date, 26 mature miRNAs encoded by HCMV (Figure 1) have been identified to target viral and host cellular transcripts to potentially evade immune defense and control cell cycle and vesicle trafficking [9,10,11,12,13,14,15].

HCMV miRNAs are differently expressed during latent and lytic infection [16]. Of note, most of the miRNAs produced during viral latency originate from the unique long (UL) region of the HCMV genome [16]. All known viral miRNAs, including those that are absent during latency, restore their expression in the reactivation of the virus from latency. Interestingly, the two strands of HCMV miRNAs are expressed alternatively during the lytic and latent viral life cycle. Specifically, miR-US29-3p dominates viral latency, whereas miR-US29-5p prevails during lytic infection [16].

The role of HCMV miRNAs and their potential mechanism of infection have been recently reviewed by Zhang [17] and Piedade and Azevedo-Pereira [18]. Currently, seven HCMV-encoded miRNAs have been reported to target potential mediators of immune evasion to improve viral replication. miR-UL112-3p, one of the first cloned HCMV miRNAs [19], binds the 3′UTR of MHC class I polypeptide-related sequence B (*MICB)*, a stress-induced ligand for the natural killer (NK) cell activating receptor NKG2D [20], leading to decreased binding of NKG2D and reduced killing by NK cells [21]. miR-UL112-3p inhibits NK cell activity by targeting other transcripts, including interleukin-32 (IL-32), type I interferon (IFN-I) signaling, and the pattern recognition receptor toll-like receptor 2 (TLR2) [22]. miR-UL148D is one of the most highly expressed viral miRNAs during latent infection [23]. This miRNA contributes to immune evasion by targeting the regulated upon activation normal T cell expressed and secreted (RANTES), a chemokine known to attract immune cells to sites of inflammation and causing tissue damage [23]. Interestingly, treatment with a miR-UL148D-specific inhibitor reverted the downmodulation of RANTES, supporting a role of this agent as therapeutic tool against HCMV infection [23]. miR-UL148D is also able to target the activating receptor type-1B (ACVR1B) in monocytes, resulting in a reduced secretion of IL-6 [24]. miR-UL22A-5p and miR-UL22A-3p play a significant role in immune modulation [17]. miR-UL22A-5p has been predicted to target bone morphogenetic protein receptor type 2 (BMPR2), a receptor involved in alternative polarization in macrophages [25]. In addition, it is also able to target heat shock 70 kDa protein 8 (HSPA8), a crucial molecular regulator belonging to the heat shock proteins (HSP70) family that induces the immune clearance of viral replication in transplant recipients through the expansion of HCMV-specific T cells [25]. In addition, miR-UL22A-5p and miR-UL22A-3p have been found to target small mother against decapentaplegic 3 (SMAD3), a mediator of transforming growth factor β (TGF-β) signaling, in infected CD34^+^ hematopoietic progenitor cells to maintain latency [26]. miR-UL59 has been shown to contribute to immune evasion by targeting UL16 binding protein 1 (ULBP1), a molecule that mediates the immune elimination of HCMV-infected cells by NK cells [27]. Finally, two HCMV microRNAs, miR-UL112-5p and miR-US4-1, were found to contribute to immune evasion by directly targeting the endoplasmic reticulum (ER) aminopeptidase 1 (ERAP1), a key component of the antigen processing that generates antigenic peptides for presentation to cytotoxic CD8^+^ T lymphocytes and NK cells [28,29]. The reduced trimming due to HCMV-specific action led to the reduction of HCMV-specific CD8^+^ T cell responses [28,29].

*ERAP1* is expressed in all individuals and displays a significant degree of polymorphism that affects the enzymatic activity and/or the expression level [30]. Natural *ERAP1* variants include functional non-synonymous single nucleotide polymorphisms (SNPs) that have been associated with predisposition to several diseases, including autoimmune diseases, virally induced cancer, and viral infections. SNPs are the largest source of sequence variations in humans. Most SNPs have no effect on health or development, whereas others have been proven to be very important in predicting an individual response to certain drugs, or the development of a myriad of diseases including susceptibility to viral infections. SNPs may be located in both the coding and non-coding regions of the genome, with a frequency of at least 1% in a random set of individuals in a population. Interestingly, SNPs occurring within miRNA-binding sites by affecting the miRNA-target interaction may deeply alter the expression of target genes. This review will focus on the relevance of *ERAP1* SNPs within miRNA binding sites in modulating viral miRNA–mRNA interactions and the possible consequent individual susceptibility to HCMV infection.

## 2. Biological Functions of ERAP1

**Figure 2:**
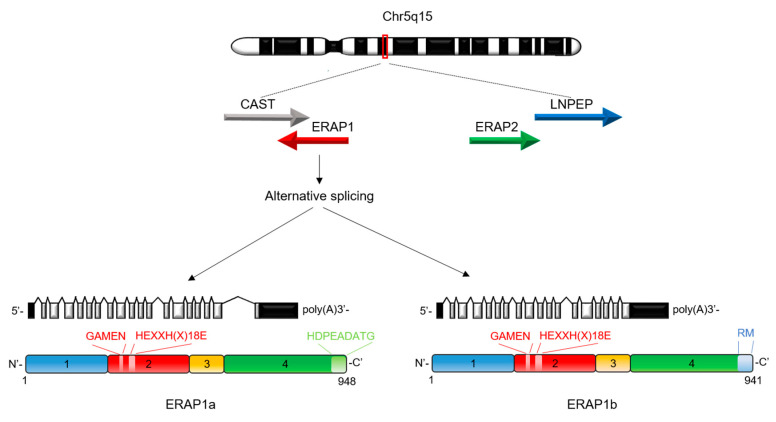
Genomic organization of the human endoplasmic reticulum aminopeptidase 1 (*ERAP1*). Genomic organization of the human chromosome 5q15 containing the *ERAP1* gene. Exons are depicted as boxes. Alternative splicing of the *ERAP1* gene gives rise to two isoforms, ERAP1a and ERAP1b of 948 and 941 amino acids, respectively. The crystallographic structure of human ERAP1 contains four domains indicated with different colors. The GAMEN and HEXXHX (18) E motifs are highlighted in domain II. The amino acids that differentiate the two isoforms at 3’untranslated region (3′UTR) are indicated.

ERAP1 is a member of the oxytocinase subfamily of M1 zinc metallopeptidases with which it shares two key sequence motifs essential for their enzymatic activity, HEXXH(X)_18_E zinc-binding and GAMEN substrate recognition sequences [31]. In humans, the *ERAP1* gene is located on chromosome 5q15 in a 167 Kb segment in the opposite direction of its homologous *ERAP2* from which it arose by gene duplication [32] (Figure 2). Alternative splicing in the *ERAP1* gene results in the formation of two N-glycosylated isoforms, namely, *ERAP1a* (20 exons and 948 amino acids) and *ERAP1b* (19 exons and 941 amino acids), which share the same amino acid sequences except for some amino acids at the C-terminal and several 3′-UTR sequences [33]. The *ERAP1b* isoform is the most expressed of the two isoforms and the only one to be targeted by HCMV miR-US4-1 and miR-UL112-5p [28,29].

ERAP1 plays an important role in various biological processes that require the trimming of amino acid residues at the N-terminal polypeptide level by picking the substrates based on both N-terminus and internal sequences [31]. Hydrophobic amino acid residues (with the exception of proline) and peptides with a hydrophobic C-terminus are preferentially cleaved by ERAP1. The major histocompatibility complex (MHC) class I immunopeptidome is shaped by the enzyme in the ER, by trimming the N-terminal residues of antigenic precursor peptides, to produce peptides of optimal length (8–10 mers) to bind the MHC class I groove. MHC class I binding peptides are generated in the cytosol mainly by the proteasome with the C-terminus suitable to bind MHC class I molecules, and then transported into the ER by the transporter associated with antigen presentation (TAP) to be trimmed at the N-terminus by ERAP1 and its homologous ERAP2. The antigenic peptides with the correct length and sequence bind MHC class I molecules. The resulting peptide–MHC (pMHC) class I complexes are then transported on the cell surface for recognition by NK cells and cytotoxic CD8^+^ T cells. Studies in animal models have revealed that downmodulation of ERAP1 significantly reduces MHC class I expression at the cell surface up to 70%, depending on the MHC class I allele [34,35]. Consistently, immunopeptidome analyses have revealed that *ERAP1* knockout cells show both quantitative and qualitative differences in the peptide repertoire as compared to *ERAP1*-expressing cells [30]. Two hypotheses have been formulated to explain how ERAP1 trims precursor peptides. In the “molecular ruler” hypothesis, ERAP1 trims free peptides [36], whereas in the “MHC class I template” hypothesis, the MHC class I molecules act as templates for ERAP1 trimming, stably binding the peptides trimmed by ERAP1 when they reach the correct length [37,38,39]. The crystal structures of ERAP1, solved by different authors, revealed a four-domain structure enclosing a large internal cavity containing the catalytic zinc ion [40,41] (Figure 2). Domain II contains both catalytic zinc and the HEXXH and GAMEN motifs. Domains I and IV enclose the cavity of the active site: domain I has a cap at the end of the amino terminal of the site and domain IV a bowl that constitutes most of the effective cavity. Domain III is a β-sandwich structure, linking domains II and IV. ERAP1 protein seems to adopt two distinct conformations (named as open and closed conformations). Structural comparison between the two conformations suggest that the open conformation is the peptide-receptive state of ERAP1 that is able of binding the peptide, whereas the closed one is the active form [41]. At the present time, no crystal structures having a peptide bound in the active site of ERAP1 have been resolved. 

ERAP1 is also involved in other immunological functions, such as (i) the regulation of innate immunity and inflammation, by boosting the shedding of cytokine receptors, such as IL-6 receptor α, type II IL-1 decoy receptor, IL-1β receptor, and TNF receptor 1 [42,43]; (ii) induction of NK cell development and function [44]; (iii) the nitric oxide synthesis [45]; and (iv) activation of phagocytic activity of antigen-presenting cells such as splenic dendritic cells (DC) and macrophages [46]. Furthermore, ERAP1 has also been associated with non-immunological functions, such as the regulation of blood pressure [47] and post-natal angiogenesis processes [47], as well as promoting Hedgehog pathway-dependent tumorigenesis by regulating ubiquitin specific peptidase 47 (USP47) and enhancing proteasomal degradation and ubiquitylation of β-transducin repeats-containing proteins (β-TrCP) [48]. 

According to these multiple functions, ERAP1 is also referred to as puromycin-insensitive leucine-specific aminopeptidase (PILS-AP), adipocyte-derived leucine aminopeptidase (A-LAP), aminopeptidase regulating tumor necrosis factor receptor 1 (TNFR1) shedding (ARTS-1), and endoplasmic reticulum aminopeptidase associated with antigen processing (ERAAP). Herein, we will refer to it as ERAP1, the official name accepted by the Hugo Gene Nomenclature Committee (HGNC). 

Expression of *ERAP1* is regulated at both transcriptional and post-transcriptional levels. Like the other components of the antigen processing, *ERAP1* is induced by Interferon γ (IFNγ) and Tumor Necrosis Factor α (TNFα) [49,50]. In addition, four microRNAs were identified as affecting *ERAP1* expression in different human disorders. An extensive microRNA profiling in patients with Behçet’s disease (BD) revealed that miR-330 and miR-181a-5p target *ERAP1* during its active phase [51,52]. In type I diabetes, *ERAP1* was found to be regulated by the IRE1a/miR-17-5p axis, thus suggesting its involvement in β-cell destruction [53]. In addition, miR-294 has been revealed to bind *ERAP1*, contributing to the reprogramming of fibroblasts to pluripotent stem cells [54].

## 3. Functional Consequences of *ERAP1* Polymorphisms in Human Diseases

*ERAP1* is highly polymorphic, with variants that alter the peptide trimming activity, specificity, and expression, even if they reside far from the active site region. Several *ERAP1* genetic variants have been associated with multiple human leukocyte antigen (HLA) class I autoinflammatory disorders, including ankylosing spondylitis (AS), BD, psoriasis, multiple sclerosis (MS), and type I diabetes, as well as essential hypertension and susceptibility to infectious disease, such as human papilloma virus (HPV)-induced cancer, HIV, hepatitis C virus (HCV), and HCMV infection [55,56,57]. In the context of autoimmune diseases, these genetic changes contribute to immune dysregulation in individuals with a specific HLA class I background [57]. Indeed, changes in the immunopeptidome due to *ERAP1* SNPs are exacerbated in the individuals carrying HLA class I loci conferring risks to disease—HLA-B27 for AS, HLA-Cw*06 for psoriasis, and HLA-B*51 for BD. Recently, *ERAP1* SNPs have been found to form multiple combinations (13 allotypes) within the populations with AS with composite functional properties that further increase the risk of disease [57,58]. 

The role of ERAP1 in modulating susceptibility to infections from HCV, HCMV, influenza virus, HPV, HIV, and toxoplasma has been exhaustively reviewed by Saulle et al. [59] and Yao et al. [60]. Several *ERAP1* variants affecting disease susceptibility and/or progression have been identified. They were located both in coding (rs30187, rs27044, rs26618, rs10050860, rs26618, rs26653, rs17481856) and non-coding regions, including intronic (rs149481, rs27042, rs149173, rs17481856) and 3′UTR (rs17481334) regions. Genetic variants in the *ERAP1* coding region—rs27044, rs26653, rs10050860, rs26618, rs26653, rs17481856, and rs30187—have been associated with susceptibility to HIV, HCV, HPV, and/or toxoplasmosis infection [59,60]. The intronic variants rs149481, rs27042, rs149173, and rs17481856 have been associated with susceptibility to *Toxoplasma gondii* infection, Kawasaki disease, chronic hepatitis C, and HIV [59,60]. Interestingly, most of these SNPs have also been associated with essential hypertension and autoimmune diseases, including AS, MS, psoriasis, and BD [56,59,60].

Of note, *ERAP1* SNPs may be functional also when located in non-coding regions. Romania and colleagues showed that rs17481334-G SNP variant, naturally falling within *ERAP1* 3′UTR, prevents *ERAP1* degradation by disrupting the consensus sequence for HCMV miR-UL112-5p binding [29]. Accordingly, *ERAP1* expression was significantly decreased, but only in fibroblasts from individuals carrying the AA genotype. Conversely, fibroblasts from individuals expressing the GG genotype did not show any alteration in RNA and protein levels [29]. Of note, individuals with GG genotype suffering from MS, a disease model in which HCMV infection is negatively associated with adult-onset disorder, have a significantly reduced HCMV seropositivity [29].

## 4. In Silico Prediction of miRNA-SNPs Affecting HCMV miRNA Binding Sites

Due to the conservation of the miRNA target sites, single nucleotide changes in the miRNA binding sequence on target genes may disrupt the miRNA–target interaction or create a new one [61]. For this reason, naturally occurring SNPs in the 3′UTR of target genes are candidates for functional variation that may be of interest for biomedical applications and evolutionary studies [61]. In this context, given the strong evidence obtained from two independent groups [28,29], the identification of additional SNPs within the viral miRNA binding site in the target *ERAP1* gene can be a promising strategy to shed further light on HCMV immune evasion mechanisms. 

**Figure 3:**
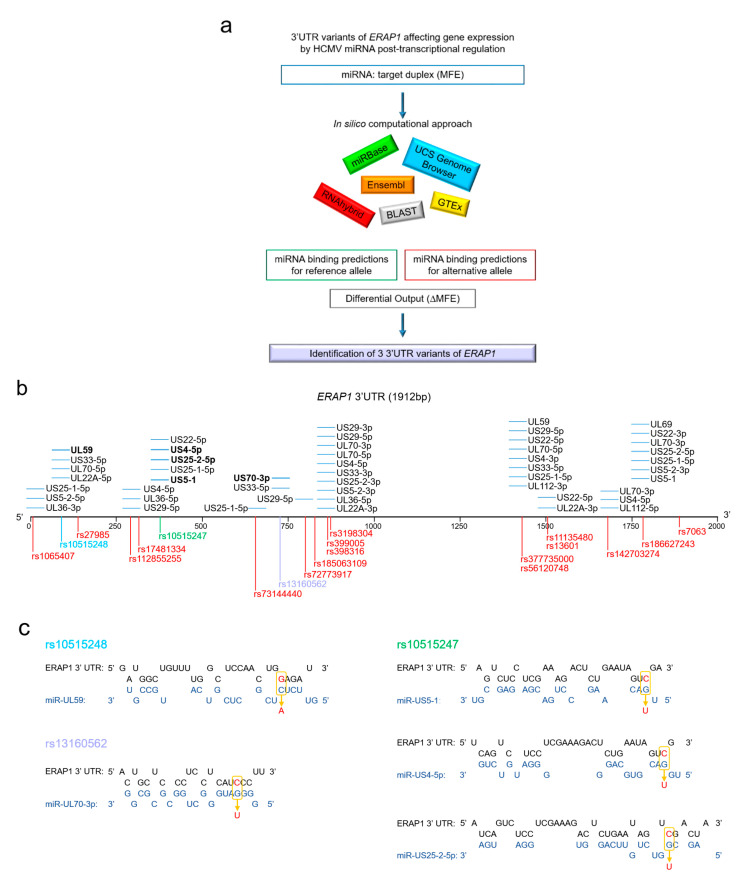
Naturally occurring *ERAP1* single nucleotide polymorphisms within HCMV miRNA binding sites. (**A**) Workflow analysis. (**B**) Genetic variants in the 3′UTR of the *ERAP1* gene at the HCMV miRNA binding sites. (**C**) Schematic representation of the indicated HCMV miRNA binding sites in the 3′UTR of *ERAP1* wild-type and variant alleles. The variant nucleotides are indicated in red.

To identify putative miRNA SNPs located at the *ERAP1* 3′UTR, which may affect the binding of HCMV miRNA, we performed an in silico analysis using different web-based bioinformatic algorithms with default parameters (Figure 3A). The following workflow was adopted: (1) extraction of HCMV miRNAs and their sequence; (2) identification of SNPs within the 3′UTR *ERAP1* region with minor allele frequency (MAF) greater than or equal to 0.1%; (3) identification of putative HCMV miRNA-binding sites within the 3′UTR SNPs; (4) assessment of the binding free energy, expressed as minimum free energy of hybridization (MFE) for both the “wild-type” and “variant” alleles identified in point 3; (5) selection of the miRNA SNP with a variation of MFE starting from -10 kcal/mol; and (6) analysis of genotype tissue expression (GTEx) of the selected putative miRNA SNPs.

The mature 26 HCMV miRNA sequences were obtained from miRBase (http://www.mirbase.org), the most updated and comprehensive database of published miRNA sequences and annotations.

The 3′UTR region of the *ERAP1b* isoform was selected by the University of California Santa Cruz (UCSC) genome browser (http://genome.ucsc.edu). Three public databases were used to collect information on SNPs located in the 3′UTR *ERAP1b* region: National Center for Biotechnology Information (NCBI) (http://www.ncbi.nlm.nih.gov), gnomAD (https://gnomad.broadinstitute.org/), and Ensembl (http://www.ensembl.org). SNPs with MAF greater than or equal to 0.1% were considered for further analyses [62]. The selected SNPs were identified in the *ERAP1b* 3′UTR using the chromosomal location provided by Single Nucleotide Polymorphism Database (dbSNP) (http://www.ncbi.nlm.nih.gov/projects/SNP) and the UCSC genome browser. The SNPs residing at the binding sites of each selected mature HCMV miRNA were screened by widespread search using Basic Local Alignment Search Tool (BLAST) (http://www.ncbi.nlm.-nih.gov/BLAST/Blast.cgi) and BLAST-SNP algorithms (http://www.ncbi.nlm.nih.gov/SNP/snpblastByChr.html) (Figure 3B). To assess the effects of these SNPs on HCMV miRNA targeting, we queried the RNAhybrid online software (http://bibiserv.techfak.uni-bielefeld.de/rnahybrid). Specifically, the sequence of the 3′UTR *ERAP1b* and that of the single mature HCMV miRNA were provided as input for the RNA hybrid analysis. For each miRNA/target interaction, we considered several parameters: (i) complementarity sequence in the seed region (positions 2-8 nt from 5′ of miRNA); (ii) presence of any mismatch in the seed region, possibly compensated by strong binding at another point in the gene; (iii) number of HCMV miRNA nucleotides involved in the interaction with *ERAP1* 3′UTR; (iv) entropy of the HCMV miRNA–target interaction. The stringency parameters were set up for each analyzed sequence and the top hits per target with MFE < −10 kcal/mol were collected. The MFE between each HCMV miRNA and their potential target binding site was calculated for both “wild-type” and “variant” alleles. The difference in MFE between the two alleles was calculated as the variation of MFE (ΔMFE) by deducting the MFE of wild-type allele from that of the variant allele. A negative ΔMFE indicates increased binding in the variant allele, whereas *vice versa*, a positive ΔMFE indicates decreased binding in the variant allele. In addition, there are also cases in which the variant causes the loss of the binding site (Table 1).

Finally, using the GTEx portal (https://gtexportal.org/) we identified potential associations between the putative miRNA SNPs and the expression level (eQTL) of *ERAP1*, thus further confirming their functional role in the *ERAP1* gene. A total of 20 out of 666 SNPs reported in Ensembl have a MAF > 0.1% and fall in the 3′UTR region of the *ERAP1b* gene (Table 1 and Table 2).

The in silico analyses revealed that each viral microRNA is capable of targeting one or more polymorphic positions of the *ERAP1b* 3′UTR region, with different MFE, suggesting a differential regulation of gene expression. Specifically, as shown in Figure 3B, Table 1 and Table 2, the genetic variants rs3198304, rs398316, rs399005, rs56120748, and rs377735000 are the polymorphic sites mostly targeted by HCMV microRNAs. An important feature to consider in the prediction analysis is the position of each SNP relative to the miRNA sequence. Of the 145 most promising predicted interactions, in 35, the polymorphism falls in correspondence with miRNA seed sequence, while in the remaining 110, it is located outside the miRNA seed sequence (Table 1 and Table 2). When the wild-type allele is replaced with the variant one, a variation of MFE ≥ +1 and ≤ -1 was retrieved in 107 predicted interactions (Table 1), whereas a loss of miRNA interaction was detected in 66 cases (Table 2). Specifically, a ΔMFE equal to or less than -1 kcal/mol was detected in 35 predicted interactions, whereas a ΔMFE equal to or greater than +1 kcal/mol was detected in 72 other predicted interactions (Table 1). Within these groups, subsets of miRNA–target interactions with a ΔMFE more or less than 4 kcal/mol (*n* = 36 with ΔMFE ≥ +4, *n* = 1 with ΔMFE ≤ -4) most likely play a functional role (Table 1). The lowest ΔMFE (-4.9 kcal/mol) was observed for mir-US25-2-5p targeting the rs73144440 variant (Table 1). This SNP has a guanine (G) as a wild-type allele and an adenine (A) as a mutant. When G is replaced by A, US25-2-5p is expected to bind more tightly to *ERAP1b* 3′UTR, causing a decrease in MFE from -21.8 kcal/mol (G) to -26.7 kcal/mol (A) (Table 1). Conversely, the highest ΔMFE (+16.6 kcal/mol) was shown for US29-5p, targeting the genetic variant rs1866272443 (Table 1). For this miRNA–SNP interaction, the wild-type allele cytosine (C) determines a MFE of -20.8 kcal/mol, which drops to -4.2 kcal/mol in the presence of the allelic variant thymine (T) (Table 1). Moreover, since in each tissue a target sequence can bind only one miRNA at a time, the sum of all ΔMFEs could represent an additional relevant parameter to predict the importance of a SNP. We can estimate that a high value of the sum of ΔMFE of the miRNA/target duplexes related to a SNP corresponds to a better probability that the SNP is functional [63]. Thus, we calculated the |*Δ*MFE|_tot_ by adding up all |*Δ*MFE|s of each SNP [64] (Table 3). The rs377735000 (G/A) was at the top of the ranking list, with a |*Δ*MFE| of 51.1. 

A total of 16 SNPs caused the loss of interactions (*n* = 66) between 23 HCMV miRNAs and the 3′UTR of *ERAP1*, but only 6 SNPs (rs10515248, rs3198304, rs13160562, rs10515247, rs112855255, rs377735000, and rs112855255) reside within the target sequence bound by the viral miRNA seed sequence (Table 2). These SNPs are preferentially targeted by eight different viral miRNAs (miR-UL59, miR-UL70-3p, miR-US4-5p, miR-US5-1, miR-US22-5p, miR-US25-2-5p, miR-US29-5p, and miR-US33-5p) (Table 2). Interestingly, the genetic variant rs10515247 is predicted to interact with three different HCMV miRNAs (miR-US25-2-5p, miR-US5-1, and miR-US4-5p), suggesting an important role in HCMV immune evasion (Figure 3C). Of note, rs10515247 has previously been found to be associated with the risk of incident hypertension [65]. In 2011, Li et al. identified a link between HCMV infection and essential hypertension, in which another HCMV miRNA (miR-UL112) was independently found to be associated with an increased risk of hypertension [66]. Therefore, given the known role of *ERAP1* in regulating blood pressure through its involvement in the renin–angiotensin system [67], both SNPs should be investigated in relation to HCMV infection. Vijayaprakash and colleagues conducted a genome-wide association study of sustained virological response (SVR) to polyethylene glycol (PEG) interferon-α (PEG-IFN-α) and ribavirin (RBV) combination therapy in 293 Australian individuals with genotype 1 chronic hepatitis C, with the aim of identifying genetic variants associated with response to treatment against HCV [68].

Among others, a suggestive association with SVR was also observed for the genetic variant rs10515248 [68]. These data suggest the importance of knowing the host genetics for the prediction of drug response, as well as for the identification of the target gene to be investigated in the treatment of viral diseases [68]. Other studies have revealed a slight association between rs13160562 and alcohol dependence [69,70], as well as type II diabetes mellitus [71]. Through the integrative DeepSAGE and RNA-sequencing approach, Zhernakova and colleagues showed that the rs13160562 variant affects the *ERAP1* gene expression levels (expression quantitative trait loci, eQTLs) and shortens the length of its 3′UTR region [72]. In this regard, 11 of the 20 genetic variants at the *ERAP1* 3′UTR identified were shown to be functional, as revealed by the Genotype-Tissue Expression (GTEx) analysis (rs1065407, rs7063, rs56120748, rs72773917, rs13601, rs11135480, rs112855255, rs10515247, rs10515248, rs13160562, and rs17481334) (Table 4). In particular, for all SNPs, the variant alleles are significantly associated with a reduced gene expression of *ERAP1* in the muscles, lungs, whole blood, and fibroblasts (Table 4). Therefore, in line with what has been experimentally demonstrated by Romania et al. [29], it is plausible to hypothesize that HCMV miRNAs preferably exert their effects on individuals carrying the most frequent SNPs at the *ERAP1* 3′UTR.

## 5. Conclusions

To date, the clinical management of HCMV infections is still challenging, given the complex interaction between virus and host [73]. Numerous factors influence an individual’s susceptibility to HCMV infection, as well as the course of the disease. This is strictly dependent on the arsenal of host immune evasion strategies encoded by the large HCMV genome [73]. In this regard, viral microRNAs are considered important players, being implicated in the viral life cycle and the pathogenic properties of the virus [74]. The first viral miRNAs were identified in 2004 in the Epstein–Barr virus (EBV) [75], whereas those encoded by HCMV were identified in 2005 [19]. Therapeutic strategies aimed at counteracting viral miRNA action, through the administration of antago-miRNAs (complementary single-stranded oligonucleotides), may represent an interesting approach to neutralize the viral infection [76]. Furthermore, targeting viral miRNAs instead of cellular ones may decrease the side effects on humans and solve the question of site-specific/non-toxic targeted release [77]. To date, viral miRNAs represent useful biomarkers for several infection diseases. Among them, HCMV-encoded miR-US4-1 is measured in the serum of hepatitis B patients as a biomarker for treatment based on IFNα administration [78].

In order to have a complete picture on the interplay between viral miRNAs and host immunity, several pieces of evidence underline the need to better characterize the composite spectrum of host genetic determinants [79]. Indeed, many human diseases are the result of non-coding variants affecting regulatory regions. In particular, a major role of the 3′UTR region has been established, due to its miRNA post-transcriptional regulation capacity [80]. Given the key role of *ERAP1* in the antigen processing machinery and virus disease control, we focused on genetic variants residing within its 3′UTR. The choice to analyze validated SNPs, bearing information on allele frequency, enhances the genotype success rate and points out these variants as real polymorphisms [81,82]. This starting point may thus facilitate the distinction of functionally neutral variants from those that effectively contribute to infection disease.

The in silico performed analyses highlight the important role of *ERAP1* in modulating the effect of miRNAs produced by HCMV in the host. It is, however, necessary to underline that all the obtained results are useful as an initial screening to select the *ERAP1* variants potentially fundamental in modulating the HCMV immune evasion strategies. A correct experimental design is thus needed to subsequently validate the emerged most promising computational predictions.

In summary, directing further investigations to the SNPs discussed here, and deepening knowledge on how 3′UTR *ERAP1* variants may alter the mechanism of action of viral miRNAs, may be useful for the development of antiviral therapies or for the treatment of complications caused by HCMV infection.

## Figures and Tables

**Table 1:** HCMV miRNAs targeting the 3′UTR of *ERAP1* gene in polymorphic sites in which the alternative allele affects the minimum free energy of hybridization (MFE).

SNP	SNP Position	MAF *	HCMV miRNA	Seed Sequence **	MFE ^#^ (Kcal/mol)	ΔMFE ^##†^ (Kcal/mol)
rs1065407 (T/G)	5:96776379	0.174	UL22A-3p	NO	−18.1	+4.7
			UL22A-5p	NO	−14.8	+1.8
			UL70-3p	NO	−23.2	−1
			US5-1	NO	−21.6	+4.7
			US5-2-3p	NO	−15	+10.8
rs10515248 (C/T)	5:96776301	0.06	UL69	NO	−22.6	3
			UL112-3p	YES	−18.6	+1.1
			US22-3p	NO	−12.4	+3.6
			US25-2-3p	YES	−24.1	+5.4
			US29-3p	YES	−16.6	+5.4
			US33-3p	YES	−20.4	+1.1
rs27985 (A/G)	5:96776250	0.006	UL59	YES	−14.3	−1.8
			UL112-5p	NO	−12.8	+5.5
			US5-2-3p	NO	−13.7	−1.9
			US5-2-5p	NO	−10.1	+5.6
			US22-3p	NO	−17.6	−1.7
			US25-1-3p	NO	−15.7	+10.6
			US25-2-5p	NO	−21.8	−1.4
rs112855255 (T/G)	5:96776101	0.06	UL36-3p	NO	−17.9	+2.1
			UL69	YES	−16.7	−2.6
			US5-1	NO	−15.3	1
			US5-2-3p	NO	−15.1	+10.8
			US22-3p	NO	−14.7	−1.3
			US33-5p	NO	−19.6	−3.9
rs17481334 (A/G)	5:96776075	0.06	UL22A-3p	NO	−11.4	−2.1
			UL36-3p	NO	−11.4	−2.1
			UL70-3p	NO	−12.7	−1.4
			UL112-3p	NO	−13.5	−1.2
			US5-1	NO	−10.9	+7.3
			US29-5p	NO	−12.9	+2.7
			US33-5p	NO	−16.3	−1.8
rs10515247 (G/A)	5:96776022	0.06	UL22A-3p	NO	−12.2	−1.8
			UL22A-5p	YES	−11.1	−2.3
			UL69	YES	−12	+1.7
			UL70-5p	NO	−13.4	+1.9
			US5-2-3p	NO	−16.9	+4.9
			US22-3p	NO	−13.5	2
			US25-1-3p	YES	−14	+1.2
			US29-5p	YES	−14.2	−1.1
rs73144440 (G/A)	5:96775743	0.008	UL22A-3p	NO	−18.6	+2.6
			US4-5p	YES	−27	+1.6
			US5-2-5p	NO	−11.6	−1
			US22-3p	NO	−20.1	−1.2
			US25-1-3p	NO	−22.3	−1.8
			US25-2-5p	NO	−21.8	−4.9
			US33-5p	NO	−23.4	+2.7
rs13160562 (G/A)	5:96775667	0.153	US4-5p	YES	−19.4	−1.4
			US25-2-5p	NO	−21.8	−2.9
			US29-5p	YES	−16.5	+1.1
rs72773917 (A/T)	5:96775598	0.06	UL59	NO	−10.8	+7.2
			US5-2-3p	NO	−17.4	1
rs185063109 (T/C)	5:96775565	0.005	US5-2-3p	NO	−15.2	+7.4
			US25-2-3p	YES	−11.9	−3.3
rs398316 (C/G)	5:96775535	0.065	UL69	YES	−12.7	−3.2
			UL70-5p	NO	−16.4	+3.1
			UL112-3p	YES	−13.8	−3.9
			US4-3p	YES	−14.7	+3.9
			US5-1	YES	−17	+3.2
			US5-2-3p	NO	−17.2	−1.3
			US33-5p	YES	−14.1	+1.7
rs399005 (T/A)	5:96775536	0.064	UL59	YES	−16.4	+3.8
			UL69	NO	−12.7	+1.3
			US5-2-5p	YES	−14.1	−1
			US29-3p	NO	−18.3	−2.8
			US33-3p	NO	−14.2	−3.1
			US33-5p	YES	−14.1	+1.1
rs3198304 (A/G)	5:96775524	0.153	UL22A-5p	NO	−11	+6.6
			US5-2-3p	YES	−17.2	−2
rs3198304 (A/G)	5:96775524	0.153	US5-2-5p	NO	−10.1	+5.4
			US25-2-3p	NO	−16.4	−2.6
			US25-2-5p	NO	−21.1	−2.5
rs3198304 (A/T)	5:96775524	0.153	US5-2-5p	NO	−10.1	+5.4
rs56120748 (C/G)	5:96774918	0.06	UL70-5p	NO	−16.3	+4.6
			US4-3p	NO	−13.4	+2.7
			US29-3p	NO	−16	+8.2
rs56120748 (C/T)	5:96774918	0.06	UL70-5p	NO	−16.3	+5.2
			UL112-5p	NO	−13.1	+4.3
			US29-3p	NO	−16	+8.2
rs377735000 (G/A)	5:96774915	0.002	UL70-3p	NO	−18.6	+15.1
			UL70-5p	NO	−14.5	−1.8
			US4-3p	NO	−13.4	+9.1
			US5-2-3p	NO	−15.2	+2.4
			US25-1-5p	NO	−15.5	+1.4
			US29-3p	YES	−16	+8.2
			US29-5p	NO	−15.6	+4.7
			US33-5p	YES	−14.7	3
rs377735000 (G/C)	5:96774915	0.002	UL59	NO	−13.3	−2.7
			UL70-3p	NO	−18.6	+14.5
			US4-3p	NO	−13.4	+9.1
			US5-2-3p	NO	−15.2	+7.4
			US29-3p	YES	−16	+8.2
			US29-5p	NO	−15.6	+5.3
rs377735000 (G/T)	5:96774915	0.002	UL70-3p	NO	−18.6	+14.2
			US4-3p	NO	−13.4	+9.1
			US5-2-3p	NO	−15.2	+7.4
			US29-3p	YES	−16	+8.2
rs13601 (G/A)	5:96774878	0.06	UL70-3p	YES	−18.6	3
			US4-5p	NO	−22	3
			US5-2-3p	NO	−12.1	3
			US25-2-5p	NO	−17.5	3
rs11135480 (T/G)	5:96774878	0.06	UL22A-5p	NO	−10.6	−1.7
rs186627243 (C/T)	5:96774610	0.002	UL22A-3p	NO	−14.9	3
			UL59	NO	−15.7	+5.4
			US5-2-5p	NO	−15.4	3
			US25-1-3p	NO	−15.9	2
			US29-5p	NO	−20.8	+16.6
			US33-5p	NO	−16.3	+1.3

* MAF, minor allele frequency; ** single nucleotide polymorphism (SNP) position in the seed sequence; # MFE, minimum free energy; ## ΔMFE, difference in MFE between the two alleles; † values of ΔMFE ≥ +1 and ≤ −1 are shown.

**Table 2:** HCMV miRNAs targeting the 3′UTR of *ERAP1* gene in polymorphic sites in which the alternative allele prevents the miRNA interaction.

SNP	SNP Position	MAF *	HCMV miRNA	Seed Sequence **	MFE ^#^ (Kcal/mol)
rs1065407 (T/G)	5:96776379	0.174	UL36-3p	NO	−10.8
			US5-2-5p	NO	−10.9
			US25-1-5p	NO	−15.8
rs10515248 (C/T)	5:96776301	0.06	UL22A-5p	NO	−12.4
			UL59	YES	−19.3
			UL70-5p	NO	−19
rs112855255 (T/G)	5:96776101	0.06	UL36-5p	NO	−14.5
			US4-5p	YES	−16.9
			US29-5p	YES	−20.1
rs10515247 (G/A)	5:96776022	0.06	US4-5p	YES	−17.7
			US5-1	YES	−14.3
			US22-5p	NO	−14.3
			US25-1-5p	NO	−18.5
			US25-2-5p	YES	−21.4
			US33-5p	NO	−16.2
rs73144440 (G/A)	5:96775743	0.008	US25-1-5p	NO	−25.2
rs13160562 (G/A)	5:96775667	0.153	UL70-3p	YES	−26.4
			US33-5p	NO	−18.6
rs72773917 (A/T)	5:96775598	0.06	US29-5p	NO	−15.8
rs398316 (C/G)	5:96775535	0.065	UL70-3p	NO	−23.8
			US29-3p	NO	−18.3
			US29-5p	NO	−20.6
			US33-3p	NO	−14.2
rs399005 (T/A)	5:96775536	0.064	UL70-3p	NO	−23.8
			UL70-5p	NO	−16.4
			US4-5p	NO	−25.2
			US5-2-3p	NO	−17.2
			US25-2-3p	NO	−16.4
rs3198304 (A/G)	5:96775524	0.153	UL36-5p	NO	−14.8
			UL70-3p	YES	−23.8
			US4-5p	NO	−25.2
			US33-3p	NO	−14.2
rs3198304 (A/T)	5:96775524	0.153	UL22A-3p	NO	−17.5
			UL36-5p	NO	−14.8
			UL70-3p	YES	−23.8
			US25-2-3p	NO	−16.4
rs56120748 (C/G)	5:96774918	0.06	US25-1-5p	NO	−15.5
			US29-5p	NO	−15.6
			US33-5p	NO	−14.7
rs56120748 (C/T)	5:96774918	0.06	UL112-3p	NO	−13.7
			US4-3p	NO	−13.4
			US25-1-5p	NO	−15.5
			US29-5p	NO	−15.6
			US33-5p	NO	−14.7
rs377735000 (G/A)	5:96774915	0.002	UL59	NO	−13.3
			US22-5p	YES	−15.6
rs377735000 (G/C)	5:96774915	0.002	UL70-5p	NO	−14.5
			US22-5p	YES	−15.6
			US25-1-5p	NO	−15.5
			US33-5p	YES	−14.7
rs377735000 (G/T)	5:96774915	0.002	UL59	NO	−13.3
			UL70-5p	NO	−14.5
			US22-5p	YES	−15.6
			US33-5p	YES	−14.7
rs13601 (G/A)	5:96774892	0.06	US22-5p	NO	−19.1
rs11135480 (T/G)	5:96774878	0.006	UL22A-3p	NO	−19.3
rs142703274 (A/T)	5:96774718	0.001	UL70-3p	NO	−21.2
			UL112-5p	NO	−11.7
			US4-5p	NO	−17.4
rs186627243 (C/T)	5:96774610	0.002	UL69	NO	−20.9
			UL70-3p	NO	−16.5
			US5-1	NO	−16.6
			US5-2-3p	NO	−20
			US22-3p	NO	−15.5
			US25-1-5p	NO	−20.1
			US25-2-5p	NO	-22.2

* MAF, minor allele frequency; ** SNP position in the seed sequence; ^#^ MFE, minimum free energy.

**Table 3:** Sum of the absolute values of variation of MFE (ΔMFE) for each identified SNP in descending order.

SNP	SNP Position	|*Δ*MFE|_tot_ *
rs377735000 (G/A)	5:96774915	51.1
rs377735000 (G/C)	5:96774915	47.2
rs377735000 (G/T)	5:96774915	38.9
rs186627243 (C/T)	5:96774610	31.3
rs27985 (A/G)	5:96776250	28.5
rs1065407 (T/G)	5:96776379	23.0
rs112855255 (T/G)	5:96776101	21.7
rs398316 (C/G)	5:96775535	20.3
rs10515248 (C/T)	5:96776301	19.6
rs3198304 (A/G)	5:96775524	19.1
rs17481334 (A/G)	5:96776075	18.6
rs56120748 (C/T)	5:96774918	17.7
rs10515247 (G/A)	5:96776022	16.9
rs73144440 (G/A)	5:96775743	15.8
rs56120748 (C/G)	5:96774918	15.5
rs399005 (T/A)	5:96775536	13.1
rs13601 (G/A)	5:96774892	12.0
rs185063109 (T/C)	5:96775565	10.7
rs72773917 (A/T)	5:96775598	8.2
rs3198304 (A/T)	5:96775524	5.4
rs13160562 (G/A)	5:96775667	5.4
rs11135480 (T/G)	5:96774878	1.8

* |ΔMFE|_tot_, sum of all |*Δ*MFE|s of each SNP.

**Table 4:** Genotype-Tissue Expression (GETx) analysis of the functional SNPs located in HCMV miRNA target sites of the *ERAP1* gene.

SNP	Muscle Tissue	Lung Tissue	Whole Blood	Fibroblasts
rs1065407	1.3 × 10^−62^	2.3 × 10^−19^	2.9 × 10^−59^	5.2 × 10^−8^
rs7063	5.6 × 10^−76^	5.4 × 10^−32^	2.6 × 10^−81^	2.6 × 10^−13^
rs56120748	2.2 × 10^−20^	6.0 × 10^−29^	3.1 × 10^−10^	3.8 × 10^−19^
rs72773917	4.0 × 10^−20^	1.4 × 10^−29^	1.6 × 10^−10^	1.3 × 10^−19^
rs13601	1.3 × 10^−20^	1.4 × 10^−29^	2.6 × 10^−10^	5.9 × 10^−20^
rs11135480	4.0 × 10^−20^	1.4 × 10^−29^	1.6 × 10^−10^	1.3 × 10^−19^
rs112855255	4.0 × 10^−20^	1.4 × 10^−29^	1.6 × 10^−10^	1.3 × 10^−19^
rs10515247	4.6 × 10^−20^	1.3 × 10^−29^	3.5 × 10^−10^	1.5 × 10^−19^
rs10515248	4.6 × 10^−20^	1.3 × 10^−29^	3.5 × 10^−10^	1.5 × 10^−19^
rs13160562	9.8 × 10^−75^	1.4 × 10^−31^	3.9 × 10^−81^	2.9 × 10^−13^
rs17481334	4.0 × 10^−20^	1.4 × 10^−29^	1.6 × 10^−10^	1.3 × 10^−19^

## Abbreviations

HCMV	human cytomegalovirus
miRNA	microRNA
UL	unique long region
US	unique short region
ERAP1	endoplasmic reticulum aminopeptidase 1
SNP	single nucleotide polymorphisms
MHC	major histocompatibility complex
UTR	untranslated region
HLA	human leukocyte antigen
MAF	minor allele frequency
MFE	minimum free energy
eQTL	expression quantitative trait loci
